# Harnessing Nuclear Magnetic Resonance Spectroscopy to Decipher Structure and Dynamics of Clathrate Hydrates in Confinement: A Perspective

**DOI:** 10.3390/molecules29143369

**Published:** 2024-07-18

**Authors:** Maarten Houlleberghs, Sambhu Radhakrishnan, C. Vinod Chandran, Alysson F. Morais, Johan A. Martens, Eric Breynaert

**Affiliations:** 1 Centre for Surface Chemistry and Catalysis—Characterization and Application Team (COK-KAT), KU Leuven, Celestijnenlaan 200F—Box 2461, 3001 Leuven, Belgium; 2 NMR/X-ray Platform for Convergence Research (NMRCoRe), KU Leuven, Celestijnenlaan 200F—Box 2461, 3001 Leuven, Belgium

**Keywords:** nuclear magnetic resonance spectroscopy (NMR), MAS NMR, static high-pressure NMR, clathrate hydrate, confined water, in situ spectroscopy, molecular water science

## Abstract

This perspective outlines recent developments in the field of NMR spectroscopy, enabling new opportunities for in situ studies on bulk and confined clathrate hydrates. These hydrates are crystalline ice-like materials, built up from hydrogen-bonded water molecules, forming cages occluding non-polar gaseous guest molecules, including CH_4_, CO_2_ and even H_2_ and He gas. In nature, they are found in low-temperature and high-pressure conditions. Synthetic confined versions hold immense potential for energy storage and transportation, as well as for carbon capture and storage. Using previous studies, this report highlights static and magic angle spinning NMR hardware and strategies enabling the study of clathrate hydrate formation in situ, in bulk and in nano-confinement. The information obtained from such studies includes phase identification, dynamics, gas exchange processes, mechanistic studies and the molecular-level elucidation of the interactions between water, guest molecules and confining interfaces.

## 1. Introduction

Clathrate hydrates, often also referred to as gas hydrates, are crystalline solids made up of cages of hydrogen-bonded water molecules containing small non-polar guest molecules [1,2]. Often-encountered guest molecules are gases such as methane (CH_4_), carbon dioxide (CO_2_) and nitrogen (N_2_), but in more extreme pressure and temperature conditions, hydrogen (H_2_) and helium (He) can also be guests [3,4]. As these compounds are typically made under high-pressure and low-temperature conditions, in nature, they are most often encountered in deep-ocean sediments and beneath permafrost layers. Natural methane hydrates have been identified as a potential future source of natural gas. The vast reserves of methane hydrates on the ocean floors and in continental margins are estimated to incorporate up to 1.8 × 10^15^ kg of methane, constituting a substantial energy resource [5]. However, extracting methane from these ocean floor hydrates presents significant technical challenges and risks. As methane has more severe greenhouse effects than carbon dioxide, it is of the utmost importance to prevent its atmospheric release by triggering the uncontrolled decomposition of the hydrates on the ocean floor [6]. Aside from being a potential source of energy, natural gas hydrates hold promise for assisting climate change mitigation. Exchanging methane with carbon dioxide, natural gas hydrates could serve as media for carbon capture and storage (CCS), sequestering CO_2_ in a solid form on the ocean floor [7,8,9]. Despite its potential, this approach still involves considerable logistical challenges and has potential ecological impacts.

The storage and transportation of energy has been identified as one of the most eminent technological applications of synthetic clathrate hydrates [10,11,12]. With their high gas storage density, they offer high potential for storing and transporting explosive gases efficiently, and with enhanced safety, as compared to (cryo-)compressed storage and transport. Confined clathrate hydrates have recently been proposed as a central element in carbon-free energy supply chains [13,14]. Beyond energy and climate mitigation, clathrate hydrates are being explored for several innovative applications. Their unique properties could be exploited in water desalination processes [9,15,16,17], providing a new method for obtaining fresh water from seawater. Hydrates can selectively capture and store specific gases, making them useful in gas separation [18,19], for example, to remove CO_2_ from (bio-)methane feeds [20,21]. Their potential to revolutionize energy resources, mitigate climate change and contribute to technological advancements will keep clathrate hydrate research at the forefront of research and innovation. Understanding the formation and decomposition mechanisms of clathrate hydrates in confinement is crucial for developing effective application strategies.

Methane and CO_2_ clathrate hydrates are easily synthetized in bulk, pressurizing these gases in the presence of water close to its freezing point. Compared to CH_4_ and CO_2_, forming H_2_ and He clathrate hydrates is less evident. On Earth, these clathrate hydrates do not occur naturally. The most common crystal structures encountered in crystalline gas hydrates are designated as structure I (sI), structure II (sII) and structure H (sH), each exhibiting different types and different combinations of water cages [22,23,24]. Structure II H_2_ hydrate has been synthesized in bulk at pressures of 2000 bar, at temperatures well below the freezing point of water [25]. Depending on the structure of the clathrate phase and the potential for hosting multiple hydrogen molecules per type of clathrate cage, the hydrogen storage capacity can rise to 7.2 wt.%, rivaling state-of-the-art compression and liquefaction technologies in terms of volumetric storage capacity [4,26].

In bulk, gas hydrates typically form very slowly and in rather extreme conditions. Kinetic promoters, such as surfactants, polymers or amino acids, have been used to speed up the inherently slow formation kinetics of clathrate structures [27,28,29]. Aside from kinetic promoters, thermodynamic promoters have also been implemented in research, which aims to enable commercial gas hydrate applications [30]. Heterocyclic compounds such as tetrahydrofuran (THF), dioxolane and dioxane, as well as hydrocarbons such as neohexane and cyclopentane, influence the thermodynamic stability of a hydrate system, shifting its phase equilibrium to higher temperatures and lower pressures [31,32]. This enables researchers to synthesize and stabilize clathrates under milder conditions. However, the addition of promoters reduces the gas storage capacity, as promoters usually occupy the larger clathrate cages. One of the most recent innovations in the field involves the introduction of porous materials in a clathrate synthesis system [33,34,35]. Carefully tuned porous materials have been demonstrated as alternatives for kinetic and thermodynamic promoters incorporated into the clathrate hydrate structure. Host surface irregularity influences the local water structure, inducing heterogeneous nucleation sites, which ultimately lead to an amorphous precursor phase that helps hydrate crystallization. Additionally, the intrinsic porosity of the host facilitates the homogeneous dispersion of water and increases the gas–water interphase, improving the kinetics of formation [35]. The confinement of hydrates in pores has been proven to confer additional stability, enabling clathrate hydrate formation at milder pressures by exploiting the quasi-high-pressure effects of nano-confinement. Confinement in nano-porous materials has, therefore, been put forward as a method to stabilize hydrate structures in temperature and pressure conditions where they would be thermodynamically unstable in bulk. 

While nano-confinement alleviates pressure requirements for gas hydrate formation and enhances formation kinetics without jeopardizing overall gas storage capacity, it introduces difficulties in the physicochemical characterization of such nano-confined hydrates. Nano-confinement reduces the length scale of periodicity by multiple orders of magnitude. This renders commonly used characterization methods for the identification and structural characterization of bulk crystalline hydrate phases, mostly neutron and X-ray diffraction, largely inefficient, as nano-sized crystalline domains give rise to broad diffraction lines. This renders the extraction of precise structural information very difficult.

Despite obvious challenges, nuclear magnetic resonance (NMR) spectroscopy characterization remains robust under nano-confinement conditions. As NMR spectroscopy exploits nuclear spins to probe their local chemical environment, irrespective of the phase or local structure they are part of [36], all NMR techniques, including absolute quantification and correlation experiments in the time and frequency domains, remain useable in nano-confined environments [37,38,39,40], even at high pressures [41,42]. In contrast with X-ray diffraction, NMR spectroscopy also does not rely on periodicity. As a result, it can probe local environments and dynamics in the presence of small domain sizes and structural disorder. Its sensitivity to local chemical environments allows it to provide valuable information on the (re)arrangement of water molecules [43], phase transitions and structural changes, cage occupancy, guest–host interactions [40,44] and molecular dynamics, also in nano-confined clathrates. As it is non-destructive, NMR spectroscopy also allows for repeated measurement of the same sample under different conditions, which is particularly useful for in situ studies of dynamic processes such as clathrate hydrate formation. Alternative spectroscopies for clathrate hydrate studies include Raman and Infrared spectroscopy. Raman spectroscopy provides direct access to the vibrational state of hydrogen and water, with four specific regions of the Raman spectrum (100–5000 cm^−1^) exhibiting characteristic features of ice and gas hydrates [45]. In situ Raman spectroscopy is sensitive to phase transformations or changes in cage occupancy and can be applied in a high-pressure environment [46]. Raman fingerprints allow one to probe and identify both ortho- and para-H_2_ and the identification of various gases, hydrates and ice phases [47,48,49,50,51]. Hydration numbers and even key thermodynamic parameters such as changes in chemical potential (Δμ^0^) can be extracted [52]. Compared to NMR spectroscopy, the benefits provided by Raman techniques come at the expense of a less straightforward quantification and the inability to directly probe through-space interactions between constituents of the clathrate hydrate and confining host materials. As NMR spectroscopy excels in the latter, it is clear that NMR and Raman spectroscopy are extremely complementary and would optimally be used in parallel, within a single instrument. Such an instrument has, however, yet to be developed. Enabling simultaneous in situ NMR and Raman spectroscopy will critically depend on the development of NMR probe heads, both static and MAS, with optical fibers for Raman spectroscopy integrated in the probe. Other than the obvious difficulties encountered with optical fiber-detected Raman spectroscopy (e.g., sensitivity, focusing optics and spurious background signals), an important aspect in the integration of complementary diagnostics in NMR probe heads is the optimization of the probe head to minimize the overall perturbation to the applied magnetic field, and to establish real-time communication between the NMR spectrometer and the software driving the acquisition of the complementary metrics. Triggering external devices and pulse sequence driving the spectrometer, together with probe head optimization towards linewidth minimization, have been previously achieved and documented [53,54,55,56,57,58].

In summary, while traditional characterization techniques become less efficient for nano-confined clathrates due to decreased domain sizes and increased structural disorder, NMR spectroscopy remains a powerful tool. Its ability to probe local environments independently of the nature of the sample under observation renders NMR particularly suited for in situ studies into the complex process of hydrate formation in nano-confinement. The following sections showcase recent findings in the field of nano-confined clathrate hydrate research, highlighting key innovations in the field of NMR spectroscopy and their impact on the study of clathrate science and technology.

## 2. Sample Environments Enabling In Situ NMR Spectroscopy on Clathrate Hydrates

### 2.1. High-Pressure Environment for In Situ Static NMR Spectroscopy

**Figure 1:**
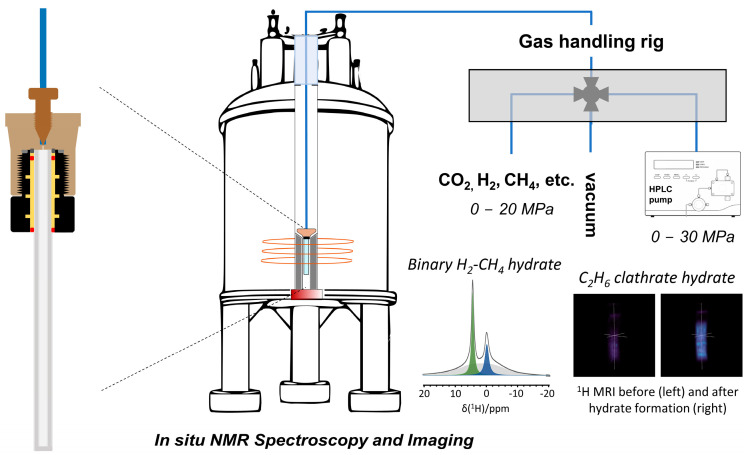
Design and assembly of a low-cost high-pressure sample cell, comprising a standard 5 mm single crystal sapphire tube retrofitted to a section of a relatively inexpensive polyetheretherketone (PEEK) HPLC column, renders in situ high-pressure magnetic resonance spectroscopy and imaging up to 30 MPa available to anyone with access to an MR spectrometer equipped with a commercial 5 mm NMR or MRI probe head. Showcase examples include the spectroscopic observation of binary clathrates occluding both H_2_ (green) and CH_4_ molecules (blue) (bottom left inset) and the visualization of C_2_H_6_ clathrate hydrate formation with ^1^H MRI (bottom right inset).

NMR spectroscopy can make use of static and magic angle spinning (MAS) approaches to study nano-confined clathrate hydrates. For solid samples, MAS NMR spectroscopy is often preferred given the increased spectral resolution under MAS conditions. However, high-pressure MAS NMR requires specific hardware, and the sample composition cannot be altered without interrupting the NMR experiment [59]. Luckily, the guest molecules possess enough rotational mobility to enable their observation with relatively high resolution without MAS. This allows one to investigate in situ clathrate hydrates, their formation, stability and decomposition under a wide range of conditions. For this purpose, a cost-effective, high-pressure sample environment has been developed [60]. This NMR tube operates at a wide range of pressures and temperatures and is compatible with standard NMR probe heads. Comprising a standard 5 mm sapphire tube (alumina single crystal) fitted to a section of polyether ether ketone (PEEK) connection compatible with standard HPLC equipment enables online studies, interfacing the cell with a gas rig and an HPLC pump (Figure 1). It is compatible with any 5 mm Broadband Observe (BBO) NMR probe head and has no significant background in NMR experiments (except ^27^Al NMR). The cell is also equipped with a safety system to handle the pressurized tube outside the probe head, ensuring the reliability and safety of high-pressure NMR experiments [60]. It enables precise control over the experimental conditions, allowing for the in situ observation of the formation of methane and ethane clathrate hydrates with NMR spectroscopy and imaging.

### 2.2. High-Pressure MAS Rotor for NMR Spectroscopy on Clathrate Hydrates

In the case of nano-confined clathrates, static NMR spectroscopy alone is often insufficient to decipher dynamic interactions between the hydrate and the confining porous host. MAS NMR spectroscopy enables the observation of the solid host fraction and the extraction of valuable information on water–surface interactions, water–guest gas interactions, guest gas–surface interactions and modifications of water properties upon interaction with the surface. MAS NMR can, for example, reveal how water molecules are structured within the confined space, how they interact with guest gases like methane or CO_2_, and how these interactions affect the stability and formation of clathrates. This information is crucial for applications such as gas storage, carbon capture and the development of advanced materials for energy applications.

**Figure 2:**
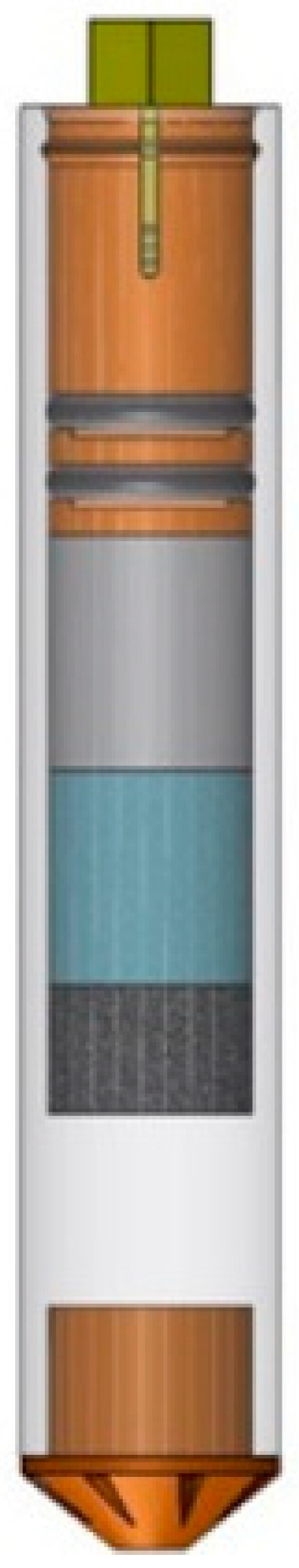
Model high-temperature/pressure WHiMS MAS rotor with a three-phase sample mixture, solid (black), liquid (blue) and gas (gray). Adapted from Ref. [59] with permission.

The application of MAS NMR spectroscopy in these studies necessitates the use of high-pressure MAS rotors, capable of handling elevated pressures and low temperatures. One such design, invaluable for nano-confined clathrate hydrate research, is the WHiMS rotor (Figure 2) [59]. Before the start of the experiment, the rotor is loaded with all solid and liquid analytes taking part in the experiment and then closed. The WHiMS rotor has a mating ridge and groove to stabilize a bushing with O-rings. The pressure of gas forces a change in the O-ring grooves in the bushing, which makes it a one-way check valve. Following closure of the rotor, this allows one to increase the pressure by loading inert or reactive gas. WHiMS rotors are designed for in situ NMR studies of reactions, with high resolution. Samples can be sealed, pressurized in a vessel and used without air exposure, allowing for sequential experiments with different gases. The rotors can withstand pressures up to 400 bar (6000 psi) for 5 mm rotors and 275 bar (4000 psi) for 7.5 mm rotors [59].

## 3. NMR Methods for Clathrate Hydrate Research

The interaction of water with nano-confining surfaces leads to a reorganization in the hydrogen bond network of water, significantly altering its properties [61]. Structurally, water molecules near confining surfaces often form distinct, ordered layers, with altered hydrogen bonding compared to bulk water and with reduced translational and rotational mobility, resulting in lower diffusion coefficients and slower relaxation. The melting and freezing points shift dramatically, and new phases can emerge. The dielectric constant decreases, and confined water can display anomalous density fluctuations and capillary effects. The degree of these changes is linked to the surface properties, and different classes of materials with a wide variety of surface chemistry, like organofunctionalized mesoporous silica, periodic mesoporous organosilica (PMO) [62,63], covalent organic frameworks (COF) [64], etc., have been shown to be efficient in altering confined water properties [61,65,66,67]. Gaining an in-depth understanding of the influence of pore chemistry and pore topology on the confined water properties, unprecedented control on the water properties by controlling the surface chemistry can be achieved, as, for example, in the so-called WaTuSo concept [61]. This requires an in-depth understanding of the material properties and especially of the interactions of water molecules with the confining material. Such information is most efficiently obtained using NMR spectroscopy.

### 3.1. Absolute Quantification

The versatility of NMR spectroscopy for molecular identification and structure elucidation of complex structures in fields ranging from pharmaceuticals to materials science is well documented. NMR spectroscopy can be used to measure relative, as well as absolute concentrations of individual components in complex mixtures, irrespective of their physical state. While relative quantities are inherently embedded in nearly every NMR spectrum acquired with direct excitation, absolute quantification requires standard addition combined with direct excitation NMR spectroscopy. In MAS probes, precise sample insertion and positioning are critical for reproducibility. Employing uniform MAS rotor filling and ensuring uniform Q-factors across the measurement range to ensure constant experimental parameters, an NMR spectrometer can be transformed into an absolute nuclear spin counter using standard addition, thus achieving absolute quantification while maintaining high resolution. This methodology has successfully been demonstrated to quantify confined water and organic solvents adsorbed in porous materials [37,38,40]. In addition to adsorbed solvents, active pharmaceutical ingredients loaded in mesoporous silica carriers and solid trace impurities in silica have also been quantified [38,68]. Also, in clathrate research, this methodology is extremely useful as it allows for quantitative analysis of the confined water phase as well as of all guest molecules in gaseous, dissolved and enclathrated states.

### 3.2. Structure Elucidation

**Figure 3:**
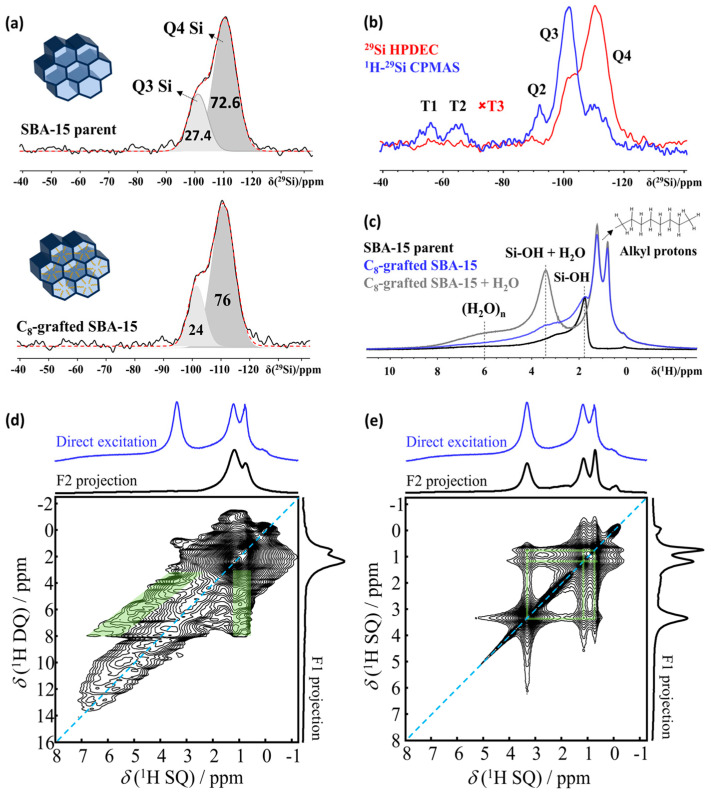
(**a**) Decomposition of the Q-part of the ^29^Si NMR HPDEC spectrum of SBA-15 material without and with C_8_ grafting (insets: schematics of SBA-15 and C_8_-grafted SBA-15). (**b**) Comparison of ^29^Si HPDEC and ^1^H-^29^Si CPMAS NMR spectra of SBA-15 C_8_. Red dashed lines in (**a**,**b**) represent the sum of the fitted Q3 and Q4 components of the spectra. (**c**) ^1^H NMR spectra of parent SBA-15 material and C_8_-grafted SBA-15 (dry and hydrated) Q_3_ and Q_4_ silicon signals are quantified with respect to their relative contributions to the total signal area of the Q-part. (**d**) ^1^H-^1^H DQSQ and (**e**) ^1^H-^1^H RFDR NMR 2D correlation spectra of C_8_-SBA-15 at 500 MHz NMR spectrometer under 15 kHz MAS. Cross correlations of rigid (H_2_O)*_n_* clusters and the alkyl protons are shown with inverted green “V” pattern (DQ-SQ), while cross correlations of dynamic H_2_O-SiOH protons and the alkyl protons are marked with green bands (RFDR).

Suitable nanoporous host materials for clathrate hydrate applications combine ordered porosity with pore walls containing patterned hydrophobic (organic) and hydrophilic (organic/inorganic) domains. C_8_-grafted SBA-15, a mesoporous silica material with cylindrical pores and post-synthetic surface modification, is an example of such a material (Figure 3a, inset). While the hydrophobic nano-confinement enforces a pseudo-high-pressure effect on the water phase diagram, the hydrophilic sections seed the nucleation of specific ice phases and impose local ordering onto the water clusters. Combined, both effects lower the overall free energy of the system, alleviating the thermodynamic requirements for the water–ice transition compared to bulk [61].

The presence of NMR active nuclei (^1^H, ^13^C, ^29^Si, ^15^N/^14^N) in nanoporous host materials of engineered pore wall chemistry makes NMR spectroscopy the prime technique for structure elucidation. Figure 3 shows what can be achieved, using the example of SBA-15 (a mesoporous silica material) grafted with C_8_ alkyl groups to tune the material properties and improve their suitability as hosts for clathrate hydrates [34]. The ^1^H MAS NMR spectrum of this nanoporous sample (Figure 3c) reveals alkyl ^1^H resonances within a range of 0.5 to 2 ppm, SiOH resonances from 1.8 to 2.2 ppm and broad resonances up to 10 ppm, indicating the presence of strongly bound water/silanol groups (denoted as (H_2_O)n). Introducing a minimal amount of water, just 2.5% of the pore volume, leads to the disappearance of silanol signals as the water molecules enter the pores and engage in rapid chemical exchange with the silanols, forming a distinct resonance at 3.4 ppm (SiOH + H_2_O). Concurrently, the intensity of the broad resonances around 6 ppm, which represent tightly bound water clusters (H_2_O)n, increases, as shown in Figure 3c. A decrease in Q_3_ Si in favor of Q_4_ Si (Figure 3a), coupled with the appearance of alkyl resonance in ^1^H NMR spectra (Figure 3a, blue trace), further confirms the grafted C_8_ chains are mainly anchored in the mesopores. Since the number of Si with grafting is very low, combined with low natural abundance, the observation of T-Si atoms in ^29^Si NMR is difficult. This can be resolved by implementing polarization transfer experiments such as ^1^H-^29^Si CPMAS NMR (Figure 3b) to selectively enhance the intensity of the resonances associated with these silicon atoms.

The arsenal of solid-state NMR pulse sequences offers a score of spin editing options to selective extract information on specific processes and species. This provides opportunities to elucidate the influence of the surface chemistry of the nano-confining host on the formation and stability of clathrate hydrates. The proximity of host–guest, host–graft and guest–graft pairs can, for example, be determined using 2D homonuclear and heteronuclear correlation NMR spectroscopy, employing J-coupling (through-bond) and dipolar coupling (through-space). Double Quantum–Single Quantum spectroscopy (DQ-SQ), for example, is adept at detecting the close proximity between different nuclei [69], and in this case (Figure 3d), it revealed the close spatial association between the grafted C_8_ chains and strongly adsorbed water molecules within the pores (Figure 3d) [34]. The characteristic inverted “V” pattern [70] observed in the 2D DQ-SQ spectrum indicates a network of spatial correlations between the strongly hydrogen-bonded water clusters (H_2_O)n and the alkyl chains, demonstrating these C_8_ chains predominantly reside within the mesopores, as opposed to being grafted on the external surface. Radio-Frequency-Driven Recoupling (RFDR) experiments [71,72] further support this conclusion by revealing the spatial proximity between the mobile water fraction and both the C_8_ chains and the strongly adsorbed water clusters (Figure 3e).

Synthesizing clathrate hydrates with D_2_O instead of H_2_O provides an extra probe nucleus, ^2^H (spin 1), adding options to, in a straightforward way, reveal the rotational mobility and, thus, the phase of water molecules in the system. ^2^H solid echo (quadrupolar echo) NMR is a powerful tool to discriminate crystalline ice from the less ordered or amorphous contributions. Quadrupolar double Pake patterns with specific quadrupole parameters like coupling constants and asymmetry parameters can act as measures for different crystalline contributions [34,73]. Introducing ^2^H via the water phase only also provides options to selectively investigate the interactions and dynamics of the water molecules in the system, as all ^2^H nuclei derive from water.

### 3.3. In Situ Nuclear Magnetic Resonance—Dielectric Relaxation Spectroscopy

**Figure 4:**
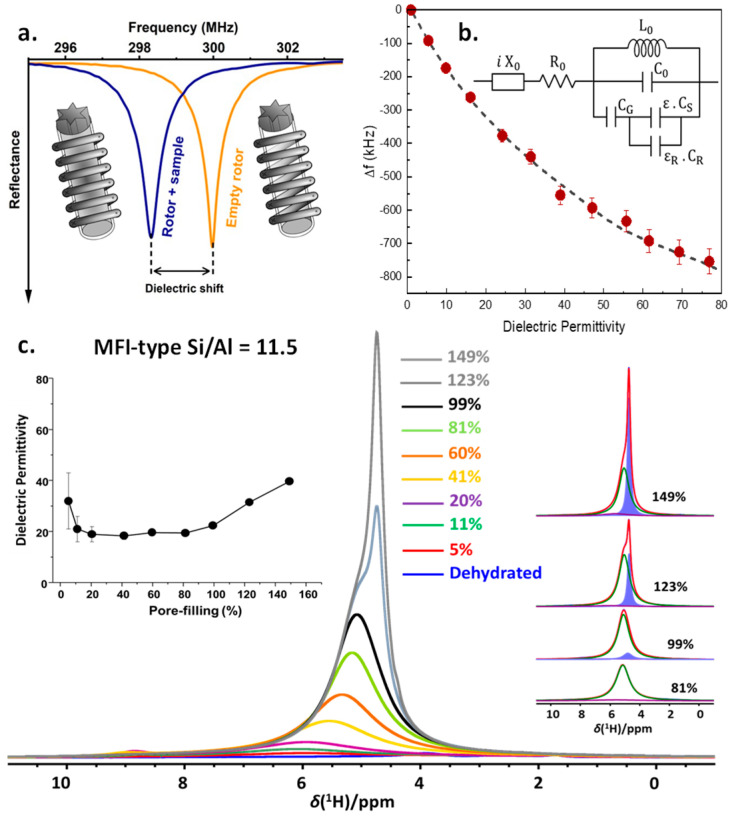
(**a**) Schematic representation of a wobble curve and the detuning (dielectric shift) upon sample insertion; (**b**) dielectric shift (Δf) shown as a function of the average dielectric permittivity (ε) of the sample in the RF coil of a 300 MHz Bruker probe head using an NMR rotor with different amounts of water. The dashed lines show the fit to the equivalent resonant circuit (inset) used to model the frequency of the NMR probe; (**c**) ^1^H MAS NMR spectra of the MFI-11.5 zeolite with different water loadings shown in percentage of pore-volume filled. The insets show the average dielectric permittivity of water in the sample as a function of the degree of pore filling (**left**) and spectral decomposition of ^1^H MAS NMR spectra at 81, 99, 123, and 149% pore filling (**right**). Adapted with permission from [74]. Copyright 2024 American Chemical Society.

To investigate nano-confined water and confined clathrate hydrates, we very recently developed a multi-diagnostic technology, enabling in situ NMR spectroscopy while simultaneously also detecting changes in the sample impedance, using the NMR coil as a remote detector circuit [74]. This allows one to very sensitively detect any change in phase or hydrogen-bonding networks of water. The technique relies on the phenomenon of the detuning of the probe upon introduction of a dielectric material into the RF coil. The magnitude of the probe detuning, referred to as “dielectric shift”, is directly correlated to a sample’s dielectric characteristics (Figure 4a). By calibrating the probe head to a sample with known dielectric properties, dielectric permittivity measurements can be performed on both liquid and solid samples in the frequency range of the NMR probe head, transforming it into a multi-diagnostic tool (Figure 4b). In this multi-diagnostic combination, high-resolution solid-state NMR spectroscopy provides molecular-level information on water dynamics and intermolecular interactions with other (water) molecules and with the confining host material. Simultaneous EIS yields more information on the nature of confined water and phase transitions. This multi-diagnostic approach is then correlated to the molecular-level elucidation yielded from NMR. This in-house-developed multi-diagnostic method has been demonstrated in static and MAS conditions for water, alcohols, as well as nano-confined water in zeolites (Figure 4c). In the case of MFI-type zeolite of Si/Al = 11.5, up to water loadings around 80% of the pore volume, average dielectric permittivity values of around 19 are observed, showing the impact of nano-confinement on the dielectric permittivity of liquids [74]. The strong surface solvent interactions in the vicinity of the adsorption sites (SiOH, AlOH, Bronsted acid sites in the case of zeolites) imposes restrictions in the geometric reorganization of water molecules, leading to a decrease in the polarizability of the water molecules involved in the interaction. Above 80% water loading, a consistent increase in the mobile water fraction is observed (inset, Figure 4c, blue component), concurrent with an increase in the permittivity. Thus, similar to what is possible using dielectric relaxation spectroscopy (DRS), the time domain version of Electrochemical Impedance Spectroscopy (EIS), this enables one to obtain data reflecting the properties of the confined water phase and its phase transitions. This recently developed technique will become a valuable tool in understanding the water organization under nano-confinement and its changes upon modification of the host material, probing in situ clathrate dynamics, furthering the understanding of the promotional effect of the confinement and contributing to the development of potential methods to tune it.

## 4. Showcase Studies

### 4.1. In Situ Static High-Pressure NMR Spectroscopy during Clathrate Formation in Bulk and under Confinement

**Showcase study 1.** In situ NMR spectroscopy during CH_4_ + THF hydrate formation in bulk

**Figure 5:**
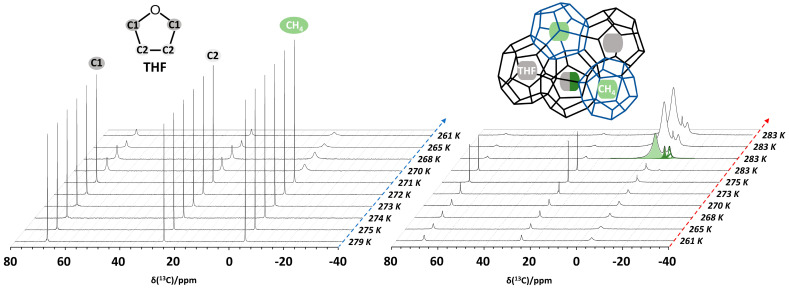
Variable-temperature ^13^C NMR spectra of the D_2_O + THF + CH_4_ system at 6 MPa methane pressure as the system evolves during cooling (**left**) and heating (**right**) cycles. An equilibration time of 5 min was used at each temperature between measurements. The ^13^C resonances corresponding to THF and CH_4_ are colored for clarity. The ^13^C resonances near −4.3 (light green), −8.5 (dark green) and −10.4 ppm (dashed green) belong to CH_4_ in the small (blue cages, inset) and large cages (black cages, inset) of the clathrate, together with the methane gas in between porous clathrate hydrate phases, respectively.

A demonstration of the capabilities of high-pressure static NMR spectroscopy for the in situ monitoring of binary CH_4_ + THF clathrate hydrate formation in bulk (5.56 mol% THF solution) is provided in Figure 5 [60]. As the temperature is gradually decreased from 279 to 261 K, the static ^13^C NMR spectra evolve, as shown in Figure 5 (left). Initially (279 K), three distinct ^13^C resonances are present. The signals at 68.2 and 25.4 ppm derive from dissolved THF, and the resonance at −4.4 ppm identifies dissolved methane. In the solution state, molecular tumbling of the solutes in water averages out intermolecular interactions, yielding sharp signals. However, at 270 K, water starts to freeze, and THF and CH_4_ becomes less mobile, leading to the broadening of the ^13^C resonances assigned to these components. At 261 K, this effect becomes more pronounced. The right pane in Figure 5 shows the evolution of the ^13^C NMR spectrum of the same sample upon increasing the temperature from 261 to 283 K. Sharp signals only start to reappear once the temperature rises above 273 K and the ice starts to melt. But the D_2_O + THF + CH_4_ system does not go back to the initial state. After ±30 min at 283 K, some broad, new resonances suddenly show up at −4.3 (light green), −8.5 (dark green) and −10.4 ppm (dashed green), while sharp ^13^C resonances attributed to dissolved THF mostly broaden and shift to 68 and 26 ppm, respectively. Keeping the sample at 283 K, the new signals at negative chemical shift keep growing. This peculiar behavior indicates the formation of clathrate hydrates occluding both THF and CH_4_. As the clathrate grows, more and more CH_4_ migrates from the headspace of the sample tube into the volume detected by the NMR coil. This process is associated with a pressure drop of 0.4 MPa, which can simultaneously be recorded by the manometer on the gas rig connected to the high-pressure cell via PEEK capillary tubing (Figure 1) [60]. The observed ^13^C chemical shifts of 68, 26 and −4.3 ppm, respectively, correspond to the two carbon sites of THF contained in the large cages of a structure II (sII)-type hydrate and to CH_4_ residing within the small cages of the same hydrate [22,75]. The ^13^C resonance at −10.4 ppm is then tentatively assigned to CH_4_ gas present in interparticle spaces and channels created as a result of clathrate hydrate formation [76].

**Showcase study 2.** In situ NMR spectroscopy of CH_4_ hydrate formation in confinement

**Figure 6:**
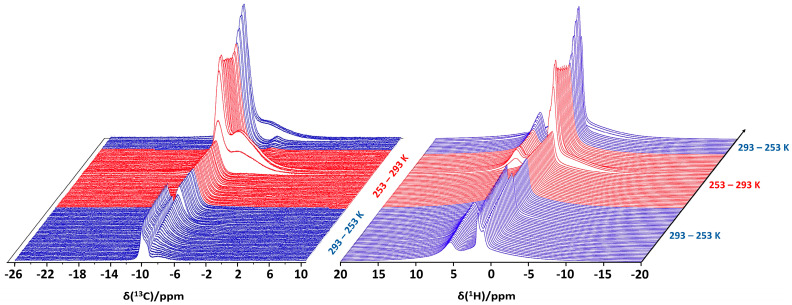
Evolution of the ^13^C (**left**) and ^1^H NMR spectrum (**right**) of D_2_O + 6 MPa CH_4_ + reversed-phase silica host as function of time and temperature. The spectra were acquired in an 800 MHz Bruker Avance Neo spectrometer in a 5 mm BBO probe head.

High-pressure static NMR spectroscopy can also be used for in situ investigations of clathrate hydrate formation in confinement. In our experiments, reversed-phase silica gel particles were packed in the high-pressure sapphire NMR tube (see Figure 1), followed by the addition of deuterated water (D_2_O) on top of the sample column. Pressurizing the tube to 6 MPa with methane gas, the deuterated water penetrated the interstitial spaces and the pores of the silica host. The sample tube was subsequently isolated from the gas supply and cooled to 253 K, re-heated to 293 K and subsequently cooled a second time to 265 K, while simultaneously recording ^13^C and ^1^H NMR (Figure 6). In the presence of the porous silica material, the ^13^C NMR spectrum is initially dominated by gaseous CH_4_ species around −10 ppm (Figure 6, left). As the temperature decreases, a new signal emerges around −8.5 ppm, which is ascribed to partly hydrated CH_4_ molecules in close proximity to the hydrophobized, C_8_-lined pore walls. Turov et al. (2008) were the first researchers to provide NMR evidence for the existence of such CH_4_ species in wetted porous systems [77]. These CH_4_ species persisted even at high temperatures and were believed to play an important role in initiating clathrate hydrate formation [77]. The intensity of the CH_4_ signal at −8.5 ppm increases as the temperature decreases, reaching a plateau at 265 K once the water freezes and molecular mobility is brought to a standstill. At the same time, expansion of the freezing water displaces gaseous CH_4_ molecules from the various interparticle voids and cavities, reducing the intensity of the NMR signals around −10 ppm. Subsequent heating gradually melts the ice phase. The cooling–heating pre-treatment of the system is aimed at degassing the water through a freezing–thawing cycle [78]. Degassing of the water effectively enhances CH_4_ dissolution. Since clathrate hydrate formation is essentially a crystallization process, involving nucleation and growth, a minimum concentration of dissolved CH_4_ is required before clathrate formation can take place. At 279 K, a broad resonance around −6 ppm emerges as CH_4_ clathrate hydrate is formed for the first time [79]. The clathrate phase decomposes at 283 K and re-emerges upon subsequent cooling once the temperature reaches 265 K.

The ^1^H NMR spectra (Figure 6, right) tell a similar story. The ^1^H signal originating from gaseous CH_4_ molecules, which are not accessible to water, is visible at 1.5 ppm, while the signal at 5.5 ppm can be ascribed to H_2_O [79]. The ^1^H signal at 0.7 ppm, which is attributed to the partly hydrated CH_4_ molecules in close proximity to the hydrophobic pore walls [77], is observed to grow with decreasing temperature. At temperatures below 265 K, the ^1^H signal at 5.5 ppm is no longer visible as the pore-intruded water starts to freeze, broadening its ^1^H signal beyond what is observable in these static NMR spectra. Upon freezing of the water phase, gaseous CH_4_ is displaced, reducing the intensity of its ^1^H signal at 1.5 ppm. Around 279 K, the water starts to melt, and CH_4_ clathrate formation takes place for the first time. Enclathration of CH_4_ molecules creates a broad ^1^H signal centered at 0.3 ppm. This resonance disappears as the clathrate hydrate phase decomposes at 283 K and re-emerges once the system is cooled to 265 K.

**Figure 7:**
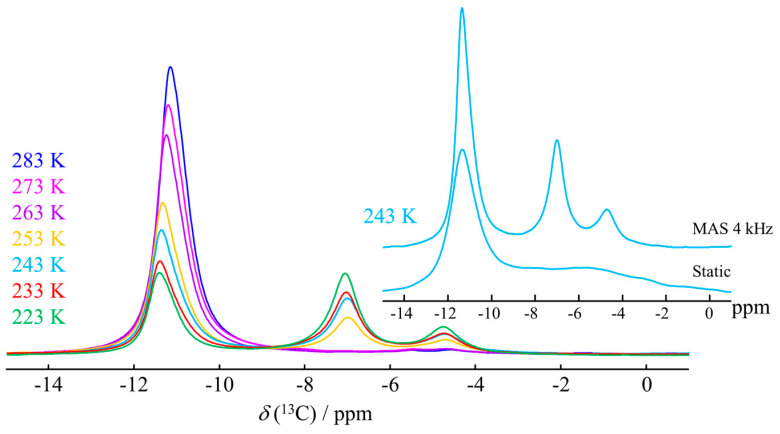
Evolution of the ^13^C NMR spectrum (right) of D_2_O + 6 MPa CH_4_ + reversed-phase silica, as function of temperature under magic angle spinning conditions. The spectra were acquired in a 400 MHz Bruker Avance Neo spectrometer, with a 5 mm MAS probe head (Phoenix NMR) and 5 mm WHiMS high-pressure MAS rotors (Phoenix NMR). (inset) Comparison of the MAS spectrum recorded at 248 K and 4 kHz MAS, with the corresponding static spectrum.

Similar high-pressure NMR experiments can also be performed under MAS conditions, rather than static. As shown in Figure 7, this yields additional resolution, albeit at the expense of experimental flexibility. In static conditions, using the high-pressure environment described in Section 2.1, both the temperature and gas pressure can be changed online during the experiment. Using the MAS sample environment (Section 2.2), the composition of the sample can no longer be changed without stopping the experiment and taking the high-pressure rotor out of the probe head. However, in addition to the resolution enhancement, using MAS NMR also provides the option to take advantage of the full set of 2D and 3D experiments available to the solid-state NMR spectroscopist. Experimental details for the results shown in Figure 6 and Figure 7 are outlined in the Supplementary Materials.

**Showcase study 3.** In situ NMR spectroscopy reveals direct exchange of CH_4_ for CO_2_ in nano-confined clathrate hydrate

**Figure 8:**
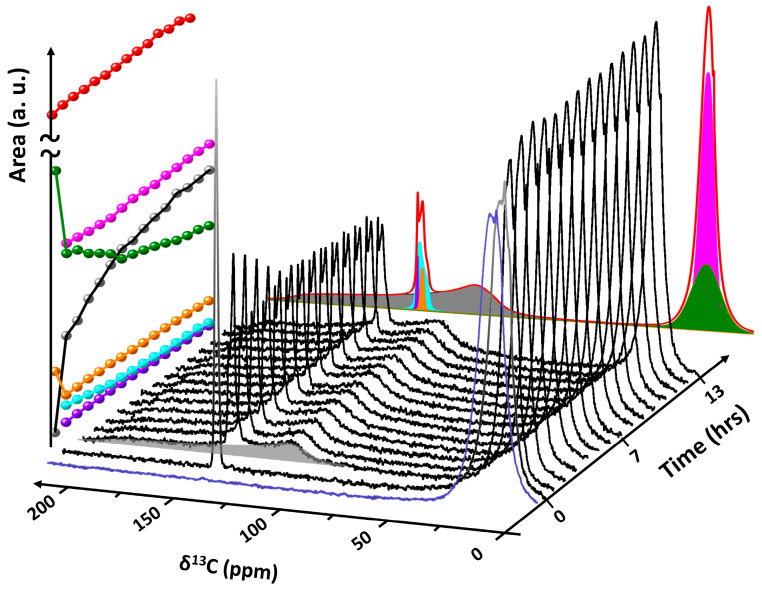
Evolution of the ^13^C NMR spectrum of confined CH_4_ hydrate (blue spectrum) upon exposure to CO_2_ gas at a total pressure of 3.5 MPa at 273 K (time ≥ 0). YZ plane: Projection of the decomposition of the final ^13^C NMR spectrum (time = 13 h). XZ plane: Plot of the signal area (a.u.) of the different spectral components as function of time (color coded for clarity: pink = gaseous CH_4_, green = CH_4_ enclathrated in sI, orange = gaseous CO_2_, gray = CO_2_ enclathrated in 5^12^6^2^ cages of sI, cyan = CO_2_ enclathrated in 5^12^ cages of sI, purple = liquid CO_2_). Red represents the total area.

Owing to their high energy density and low self-discharge, molecules such as H_2_ and CH_4_ are designated as potential long-duration energy storage (LDES) vectors, complementary to shorter-duration storage technologies such as electrochemical batteries. An important drawback of circular carbon-based energy applications is the need for expensive infrastructure for the handling of CO_2_. A safe and sustainable alternative for this would be the implementation of CO_2_ capture and storage in clathrate hydrates, initially acting as a storage material for CH_4_ and later collecting the byproduct CO_2_, which can be desorbed and transformed to methane. To validate the feasibility of this concept, an exploratory direct exchange experiment was performed using in situ static ^13^C NMR spectroscopy to monitor the system [14]. In the experiment, CH_4_ hydrate supported in hollow-ring PMO (HR-PMO) was synthesized by first hydrating the HR-PMO with ultra-pure water (Milli-Q^TM^) to a loading of 5.5 g water/g HR-PMO. Subsequently, the material was packed in a high-pressure NMR cell and pressurized with ^13^C-enriched CH_4_ gas (99% ^13^C) to 7 MPa. Transferring the cell to the spectrometer and equilibrating it for 12 h at 273 K, confined CH_4_ hydrate was formed. Via the connection to the gas rig and without removing the sample from the spectrometer, subsequently, the CH_4_ gas pressure was reduced to 0.5 MPa, followed by raising the pressure to 3.5 MPa using ^13^C-enriched CO_2_ (~50% ^13^C). The evolution of the system was monitored as a function of time using in situ quantitative ^13^C NMR spectroscopy (Figure 8). Spectral decomposition of CO_2_-exposed CH_4_ clathrate hydrate is shown at the back. Before exposure to CO_2_ gas (blue spectrum), the system exclusively contains confined sI CH_4_ clathrate hydrate (green resonance) and gaseous CH_4_ (pink resonance) [34]. Exposure of CH_4_ hydrate to CO_2_ gas at 3.5 MPa (time = 0) results in the immediate appearance of an additional ^13^C NMR associated with gaseous CO_2_ at 127.82 ppm (orange resonance). Already after 1 h, a broad feature displaying a Pake-like axial pattern emerges, centered around 127.94 ppm (gray resonance), assigned to the presence of CO_2_ molecules in the large (5^12^6^2^) oblate cages of sI clathrate hydrates [80]. At the same time, a more narrow signal appears at 128.15 ppm (cyan resonance), corresponding to CO_2_ in the small (5^12^) quasi-spherical cavities [80]. As the signal intensity for trapped CH_4_ (depicted as green spheres) diminishes over time, there is a concurrent rise in the ^13^C NMR signal linked to CO_2_ within the expansive cages (illustrated by gray dots) of sI clathrate. This suggests that there is a direct swap of CH_4_ with CO_2_, rather than the clathrate breaking down and then recrystallizing. The left plane in Figure 8 depicts the evolution of area of all resonances as a function of time. The ^13^C signal of CO_2_ molecules in small clathrate cages (cyan spheres) slightly decreases over time, evidencing the previously reported marked affinity of CO_2_ molecules for the larger 5^12^6^2^ cages of sI in combination with CH_4_ [81]. The final spectral component at 130.17 ppm (purple resonance) is very sharp, almost liquid-like in nature, assigned to the presence of a small fraction of liquid CO_2_.

**Showcase study 4.** Identification of nano-confined H_2_ + THF sII clathrate

**Figure 9:**
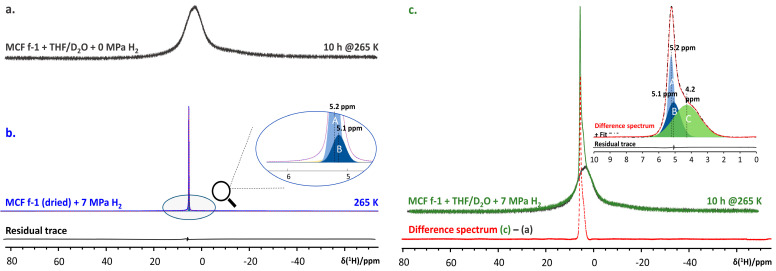
(**a**) ^1^H NMR spectrum of MCF + 5.56 mol% THF/D_2_O + 0 MPa H_2_ measured after equilibration for 10 h at 265 K (black trace). (**b**) ^1^H NMR spectrum (data, red, and fit, blue) of D_2_O-exchanged and subsequently dried MCF pressurized to 7 MPa with H_2_ gas. The residual trace is at the bottom, in black. (inset) Important spectral components ‘A’ (5.2 ppm) and ‘B’ (5.1 ppm), attributed to gaseous H_2_. (**c**) ^1^H NMR spectrum of MCF + 5.56 mol% THF/D_2_O + 7 MPa H_2_ after 10 h at 265 K (green trace). The red trace at bottom shows the difference between spectra c and a. (inset) Decomposition of the difference spectrum. Components ‘A’ and ‘B’ again indicate the presence of gaseous H_2_, while ‘C’ (4.2 ppm) is attributed to H_2_ molecules residing in 5^12^ cages of the sII clathrate hydrate. The residual of the fit is shown in black.

Mesostructured cellular foam (MCF) is a mesoporous silica, providing a continuous three-dimensional (3D) pore system with very large pores, with pore sizes even up to 22 nm [35,82]. It is exceptionally robust towards temperature and pressure swing cycles, making it a potentially attractive host material for nano-confined clathrate hydrates. Grafting promoter molecules, such as tetrahydrofuran (THF) to the MCF internal surfaces and impregnating the material with a 5.56 mol% aqueous solution of THF at 7 MPa, the material was shown to uptake more H_2_ compared to the bulk system in the same conditions [35]. Subtracting the static ^1^H NMR spectra of the MCF confined THF/D_2_O system at 265 K (Figure 9a) from that of the same system pressurized to 7 MPa using H_2_ gas (Figure 9b) readily reveals three distinct populations of H_2_ molecules in the system (Figure 9c, inset). Comparing these populations to those observed for the dry MCF material pressurized with H_2_ under the same conditions (Figure 9c) readily enables one to identify the resonance at 4.2 ppm as H_2_ encapsulated in the 5^12^ cages of sII H_2_ + THF clathrate hydrate.

### 4.2. Deciphering the Interactions Governing Nano-Confined Clathrate Hydrate Formation

An optimal material promoting the formation of nano-confined CH_4_ clathrate hydrate is touted to exhibit a mix of hydrophobic and hydrophilic functional groups, as the CH_4_ uptake of water-impregnated SBA-15 material with and without C_8_ grafting is significantly different. While both materials exhibit isotherms with similar shapes, non-functionalized SBA-15 has a significantly lower methane uptake [34]. The hypothesis that an amphiphilic material promotes the formation of confined CH_4_ clathrate hydrate can be put to test using the MAS NMR spectroscopy toolbox to identify which functional groups reside in close proximity to the enclathrated CH_4_ molecules.

MAS NMR experiments can be performed at ambient pressure by first stabilizing and preserving the hydrate structure in cryogenic conditions (liquid N_2_ temperature), subsequently releasing the excess CH_4_ gas and transferring the sample to a MAS NMR probe head pre-cooled to 223 K. For the experiment, a C_8_-grafted SBA-15 material was packed in a 7 mm zirconia MAS rotor and hydrated using D_2_O, up to a volume equal to 1.5× pore volume of the material. Subsequently, mounting a ceramic drive cap with a central opening allowed for exposure of the material in a small reactor tube pressured with CH_4_ gas at 7 MPa and thermostated at 248 K. Following incubation for 16 h, the reactor containing the rotor was flash cooled to liquid nitrogen temperature. After subsequent depressurization, the rotor was transferred into the pre-cooled (223 K) NMR probe head.

**Figure 10:**
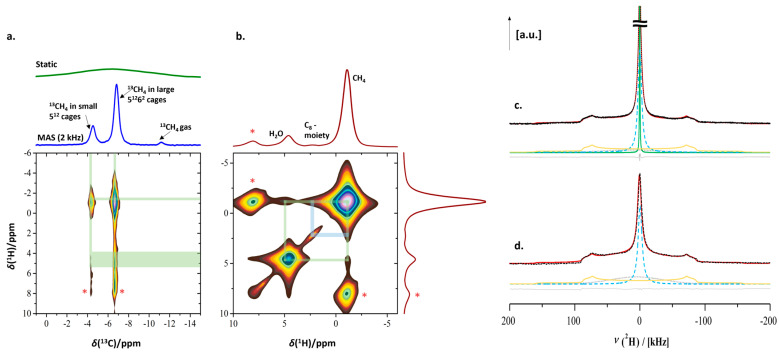
(**a**) 2D ^1^H-^13^C CP HETCOR spectrum showing the correlation of H_2_O protons and methane carbons (the asterisks represent the spinning sidebands); (**b**) 2D ^1^H-^1^H RFDR spectrum showing the spatial proximity of water protons, methyl protons and grafted alkyl groups; (**c**) ^2^H static solid-echo NMR spectrum after methane hydrate formation in wet C_8_-SBA-15 and the removal of CH_4_ at 0.1 MPa and 248 K, consisting of one additional sharp ^2^H signal (green trace) as compared to (**d**) ^2^H static solid-echo NMR spectrum of the pre-wetted C_8_-SBA-15 host material at 248 K before introducing CH_4_.

Enclathration of CH_4_ in the two distinct cages of sI clathrate hydrate was confirmed by the ^1^H-^13^C CPMAS spectrum (Figure 10a). The resonances at −4.4 ppm (^13^CH_4_ in small 5^12^ cages) and −6.7 ppm (^13^CH_4_ in large 5^12^6^2^ cages) with an integrated area ratio of 1:3, the characteristic ratio of small and large cages in sI-type clathrate hydrate [22,75], confirm this assignment. A comparison of the two 1D traces shown in Figure 10a readily reveals the dramatic effect of MAS (2 kHz) on the spectral resolution that can be obtained at these low temperatures. Interaction between ^13^CH_4_ carbon and water protons is evidenced in the ^1^H-^13^C CP-HETCOR heteronuclear correlation experiment (Figure 10a, bottom). Also, the expected correlation between the carbon and the protons of methane itself (at −1.07 ppm) is present. The interaction between the ^13^CH_4_ carbon and water protons (at 4.62 ppm) can also be observed.

The homonuclear ^1^H-^1^H RFDR correlation spectrum of the sample, run at 4.5 kHz MAS speed (Figure 10b), reveals close proximity not only between CH_4_ and H_2_O but also between the protons of enclathrated CH_4_ and the C_8_ groups grafted in the mesopores of the SBA-15 material. A correlation between the C_8_ alkyl protons and water protons was absent. Since D_2_O was used instead of H_2_O, a very small amount of residual water molecules was present in proximity with the hydrophobic C_8_ alkyl chains, making the expected correlation between the C_8_ alkyl protons and water protons below the detection limit.

Two-dimensional ^1^H-^13^C and ^1^H-^1^H correlation spectroscopy confirmed the presence of methane in an sI hydrate, inside the mesopores of C_8_-SBA-15. Combined, these spectra confirmed the promotional influence of the grafted alkyl groups on nano-confined clathrate formation kinetics.

## 5. Conclusions and Outlook

This study highlights the application of static and MAS NMR for structural analysis of clathrate hydrates in various states: bulk, nano-confined, in situ and ex situ. High-pressure sample environments for static and MAS high-pressure NMR are showcased and discussed, outlining the pros and cons of both implementations. From the showcase studies, the strength of NMR spectroscopy for clathrate research is evident, but the results also show NMR spectroscopy, by itself, cannot fully reveal all interactions in nano-confined clathrate environments. Multi-technique approaches are, therefore, crucial. Similar to NMR, X-ray scattering (SAXS/WAXS) and Raman spectroscopy are unaffected by sample scale or periodicity. Combining these techniques with NMR provides valuable insights into different aspects of confined clathrate hydrates. However, a major challenge arises when combining data: ensuring all datasets, collected separately, represent the same system under identical conditions. This is especially true for confined systems sensitive to pressure and temperature changes. Combining data from separate instruments, particularly for in situ experiments with vastly different environments, is further complicated by limited overlap between information from each technique. In such cases, verifying if samples are in comparable states becomes impossible. This is a universal issue in complex systems, requiring multiple characterization methods, not just nano-confined clathrates. Here, Electrochemical Impedance Spectroscopy (EIS) and Raman spectroscopy offer unique opportunities. The dielectric properties of water-containing samples are highly sensitive to phase changes, pressure and temperature. As shown in Section 3.3, in situ NMR/DRS allows for the simultaneous measurement of NMR spectra and sample impedance. Similarly, in situ measurement of sample impedance or conductivity can be incorporated into X-ray scattering and spectroscopy experiments, even at high pressures and low temperatures, relevant to clathrate hydrate research. While not yet demonstrated experimentally, these combined measurements are technically feasible. They present an opportunity to virtually integrate NMR spectrometers and synchrotrons using impedance as a common diagnostic tool. Recording sample impedance simultaneously with NMR and X-ray experiments would allow for a highly confident combination of results from both techniques on the same sample under identical conditions. Raman spectroscopy could play a similar role. Raman spectroscopy offers direct access to the vibrational state of hydrogen and water, revealing hydration numbers and even key thermodynamic parameters such as changes in chemical potential (Δμ^0^). Characteristic features of ice and gas hydrates allow one to fingerprint and identify various gases, hydrates and ice phases. Today, Raman spectroscopy is implemented as a simultaneous diagnostic on several synchrotron beamlines around the globe, with the Swiss–Norwegian Beam Lines (SNBL) at ESRF being a prime example [83]. Similar to the X-ray/DRS combination, combined in situ NMR/Raman spectroscopy has not been realized yet, but integration is technically feasible. Therefore, both EIS and Raman spectroscopy are valuable tools to increase confidence when combining complementary data from in situ NMR and X-ray experiments on confined clathrate hydrates.

## Data Availability

The original data presented in this study are openly available in Harvard Dataverse at https://doi.org/10.7910/DVN/X1PJLE.

## Supplementary Materials

The following supporting information can be downloaded at: https://www.mdpi.com/article/10.3390/molecules29143369/s1. The Supplementary Materials contain NMR experimental details of results shown in Figure 6 and Figure 7 presented in this review manuscript (previously unpublished results).
