# Supplementary material for: Harnessing Nuclear Magnetic Resonance Spectroscopy to Decipher Structure and Dynamics of Clathrate Hydrates in Confinement: A Perspective
Source: Molecules. 2024 Jul 18;29(14):3369. doi: 10.3390/molecules29143369 (PMC11279878; doi:10.3390/molecules29143369)
Supplement: Supplementary file 1 [file molecules-29-03369-s001.zip › molecules-3064612-supplementary.pdf]

Supporting information

Harnessing Nuclear Magnetic Resonance Spectroscopy to Decipher Structure and Dynamics of Clathrate Hydrates in Confinement: A Perspective

Maarten Houlleberghs[1,§], Sambhu Radhakrishnan[1,2,§], C. Vinod Chandran[1,2], Alysson F. Morais[1,2], Johan A. Martens[1], and Eric Breynaert[1,2,*]

[1] Centre for Surface Chemistry and Catalysis—Characterization and Application Team (COK-KAT),
KU Leuven, Celestijnenlaan 200F - box 2461, 3001 Leuven, Belgium.
[2] NMR/X-ray Platform for Convergence Research (NMRCoRe), KU Leuven, Celestijnenlaan 200F - box 2461, 3001 Leuven, Belgium
* Correspondence: eric.breynaert@kuleuven.be; Tel.: +32 16 321598

Experimental details

Figure 6: The experiments were done in Bruker 800 Neo NMR spectrometer using 5mm BBO probe. The operating Larmor frequencies of 1H and 13C were 801.25 MHz and 201.47 MHz, respectively. The sample in sapphire tube was pressurized up to 80 bar of methane. The RF strength used for 1H was 18 kHz and for 13C was 25 kHz. Power gated decoupling was used. 1H experiments used recycle delay of 10 s and 8 scans. The 13C experiments used recycle delay of 30 s and 8 scans. The resonances were referenced against those of TMS.

Figure 7: The experiments were performed using a Bruker 400 Neo NMR spectrometer in combination with a Phoenix NMR high-pressure NMR probe in combination with 5mm WHiMS high pressure MAS rotors (Phoenix NMR). The operating Larmor frequencies of 1H and 13C were 400.30 MHz and 100.66 MHz, respectively. The 5mm Phoenix rotor with sample under 60 bar of methane pressure was spun up to 4 kHz. The RF strength used for 1H was 80 kHz and for 13C was 50 kHz. 1H decoupling was achieved with SPINAL-64 sequence. 1H experiments used recycle delay of 6.2 s and 8 scans. The 13C experiments used recycle delay of 26.3 s and 20 scans. The resonances were referenced against those of adamantane.
